# A test for within‐lake niche differentiation in the nine‐spined sticklebacks (*Pungitius pungitius*)

**DOI:** 10.1002/ece3.2182

**Published:** 2016-06-14

**Authors:** Federico C. F. Calboli, Pär Byström, Juha Merilä

**Affiliations:** ^1^ Ecological Genetics Research Unit Department of Biosciences University of Helsinki Helsinki Finland; ^2^ Department of Ecology and Environmental Sciences Umeå University Umeå Sweden

**Keywords:** Ecological speciation, Gasterosteidae, habitat differentiation, niche, SNP, stickleback

## Abstract

Specialization for the use of different resources can lead to ecological speciation. Accordingly, there are numerous examples of ecologically specialized pairs of fish “species” in postglacial lakes. Using a polymorphic panel of single nucleotide variants, we tested for genetic footprints of within‐lake population stratification in nine‐spined sticklebacks (*Pungitius pungitius*) collected from three habitats (*viz*. littoral, benthic, and pelagic) within a northern Swedish lake. Analyses of admixture, population structure, and relatedness all supported the conclusion that the fish from this lake form a single interbreeding unit.

## Introduction

Environmental heterogeneity and intraspecific competition provide the opportunity and motivation for individuals to maximize their fitness by taking advantage of the resources that are most easily exploited given their specific phenotypes (Rainey and Travisano 1998; Schluter 2001; Nosil and Reimchen 2005; Reid and Peichel 2010; Siwertsson et al. 2010; Araújo et al. 2011; Faulks et al. 2015). Individuals living in sympatry can segregate into different habitats to exploit dissimilar resources; this may provide a starting point for ecological speciation, in which reproductive isolation evolves as consequence of contrasting selection pressures between different resource environments (Schluter 1996a, 2001).

Evidence for the occurrence of ecological speciation has grown over the past decades (Price 2008; Schluter 2009). Specifically, genetically divergent sympatric forms of fishes in postglacial freshwater habitats provide numerous potential examples of ecological speciation (Schluter 1996b). Well‐studied examples include different trophic forms of whitefishes (*Coregonus clupeaformis*; Bernatchez and Dodson 1990; Bernatchez et al. 1996), arctic charrs (*Salvelinus alpinus*; Hartley et al. 1992; Malmquist et al. 1992), and three‐spined sticklebacks (*Gasterosteus aculeatus*; Schluter and Conte 2009). Recently, additional examples of trophic specialization and intrapopulation divergence within relatively young Fennoscandian lakes have been emerging, including the case of Eurasian perch (*Perca fluviatilis*; Bartels et al. 2012).

Not surprisingly, most, if not all, sympatric species pairs or forms were initially discovered on the basis of phenotypic information. However, ecological speciation can also be “cryptic” and involve – at least in its initial stages – little morphological differentiation between the incipient ecotypes. For instance, behavioral differences (e.g., feeding habits, mating, and habitat preferences) in the absence of marked morphological differences could potentially render detection of such forms indistinguishable in analyses based on morphological criteria only. However, genetic methods provide means to identify possible incipient species before any clear phenotypic divergence has taken place (Bickford et al. 2007; Wiens 2007).

The aim of this study was to test for genetic differentiation in nine‐spined sticklebacks (*Pungitius pungitius*) collected from three distinct trophic environments (*viz*. littoral, benthic, and pelagic) within a single lake. The species occurs commonly in small boreal and subarctic lakes in high densities, exploiting both pelagic and benthic resources. Hence, conditions favoring divergence and ecological speciation are present (cf. Andersson et al. 2007; Svanbäck and Persson 2009). The species also shows a high degree of genetic differentiation among local populations in both marine (DeFaveri et al. 2011) and freshwater environments (e.g., Shikano et al. 2010; Bruneaux et al. 2013), but to the best of our knowledge, no study has tested for possible genetic differentiation within its freshwater isolates.

## Materials and Methods

### Samples

Fish were collected from an unnamed northern Swedish Lake (67°53′30″N, 20°05′19″E) in June–August 2002. This is a small (1.82 ha) lake situated 480 m.a.s.l. with a maximum depth of 2.5 m. The nine‐spined stickleback is the only fish occurring in this lake. Sticklebacks were collected with Ella traps (Oy Ella Fishing Ab, Hanko, Finland; mesh size 6 mm) baited with caviar. Fishing was conducted in three different zones of the lake: littoral, benthic, and pelagic. Three pairs of traps were set from the shore in the littoral zone, and two pairs of traps were each set from a boat in the benthic and pelagic zone. The captured fish were stored in 70% ethanol until the DNA extractions were made. Altogether 159 (littoral: 55; benthic: 55; pelagic: 49) randomly chosen individuals were used for genotyping.

### Resource abundance

In order to characterize resource abundance in the three habitat zones, macroinvertebrates and zooplankton were sampled in early July and mid‐August 2002. Zooplankton were sampled at four pelagic stations by hauling a 100‐*μ*m‐mesh net (diameter 25 cm) 2 m vertically from 2 m depth to the surface. The samples were preserved with Lugol's solution. Macroinvertebrates were sampled at four littoral and four benthic (offshore) stations. In the stony littoral zone, macroinvertebrates were sampled by the brushing of all organic material from three randomly picked stones (approximately 9 cm in diameter). Macroinvertebrates from the benthic habitat were sampled with an Ekman dredge. Lengths were transformed to dry mass using regressions relating body length to dry weight (Byström et al. 2007). Resource abundances (Table 1) were similar to what have been reported from other small nine‐spined stickleback lakes and from larger lakes in the same area (Johansson and Wahlström 2002; Byström et al. 2004, 2007). Dominant zooplankton taxa were calanoid copepods (78–86% by biomass) in the pelagic habitat, Trichoptera larvae and gastropods (80–90%) in the littoral habitat, and chironomids (100%) in the benthic habitat.

**Table 1:** Mean dry mass (±1 SE) of macroinvertebrates in the littoral, benthic, and pelagic habitats during in July and August sampling of the study lake

Littoral (mg m^−2^)		Benthic (mg m^−2^)		Pelagic (*μ*g L^−1^)	
July	August	July	August	July	August
198 ± 140	121 ± 50	36 ± 14	89 ± 31	31 ± 8.5	0.2 ± 0.15

### Genetic material

DNA extractions were made from fin clips of ethanol‐preserved fish in 2014 using QIAGEN DNeasy Blood and Tissue kit (QIAGEN Nordic, Sollentuna, Sweden). Approximately 5–38 ng/*μ*L of DNA per sample was used to genotype single nucleotide polymorphisms (SNPs) with the Sequenom platform (San Diego) at the Technology Centre of Finnish Institute for Molecular Medicine (FIMM). The SNPs (*n* = 66) were chosen because they were assumed to be unlinked based on the information from earlier linkage analyses (Rastas et al. 2016). Specifically, two to five SNPs per linkage group were selected in order to represent a random distribution across the genome and to maximize the probability that they were unlinked.

For genotyping, the 159 fish were split into two batches consisting of 79 (henceforth: Batch 1) and 80 individuals (Batch 2), respectively. Batch 1 was genotyped for 64 SNPs and Batch 2 for 59 SNPs. Six and two SNPs failed in all individuals in Batch 1 and Batch 2, respectively, reducing the number of useable SNPs to 58 (Batch 1) and 57 (Batch 2). A total of 55 SNPs were common among both batches. However, only 33 SNPs were polymorphic in the focal population. Of these 33 SNPs, one SNP (33347) was not in Hardy–Weinberg equilibrium (*P* = 4.8 × 10^−12^), and was eliminated from further analyses, leaving a total of 32 useable SNPs for analyses (Table 2). For these 32 SNPs, the highest pairwise *R* ^2^ between any two SNPs was 0.056, confirming that they were unlinked.

**Table 2:** Major and minor allele frequencies in 32 polymorphic SNP loci in nine‐spined sticklebacks. *P* (HWE) refers to *P*‐value of test for Hardy–Weinberg equilibrium

Marker ID	Linkage group	He	# of genotypes	Major allele (frequency)	Minor allele (frequency)	*P* (HWE)
*24644*	1	0.09	159	C (0.95)	A (0.05)	1.00
*29372*	1	0.34	157	A (0.78)	G (0.22)	0.16
*28629*	2	0.01	159	A (0.99)	G (0.01)	1.00
*29012*	2	0.49	158	C (0.57)	T (0.43)	0.87
*2438*	3	0.41	158	T (0.71)	C (0.29)	0.34
*16185*	4	0.43	159	T (0.69)	G (0.31)	0.85
*4544*	5	0.07	159	G (0.96)	A (0.04)	0.19
*17562*	5	0.15	159	T (0.92)	C (0.02)	0.31
*31597*	6	0.35	159	T (0.76)	C (0.24)	0.83
*24214*	8	0.50	158	A (0.50)	C (0.50)	0.27
*25808*	8	0.15	159	G (0.92)	T (0.08)	0.60
*29227*	8	0.47	158	T (0.62)	A (0.38)	0.18
*20626*	9	0.46	159	A (0.63)	G (0.27)	0.24
*18083*	10	0.49	159	T (0.57)	C (0.43)	0.02
*29288*	10	0.16	159	G (0.91)	A (0.01)	0.62
*19045*	11	0.01	159	A (0.997)	G (0.003)	1.00
*9694*	13	0.26	159	C (0.85)	T (0.15)	0.36
*7972*	14	0.35	159	G (0.77)	C (0.23)	1.00
*31404*	14	0.42	159	C (0.70)	A (0.30)	1.00
*12169*	15	0.47	159	C (0.62)	T (0.38)	1.00
*27696*	15	0.12	159	T (0.94)	C (0.06)	0.47
*31328*	15	0.07	159	T (0.93)	C (0.07)	1.00
*13903*	16	0.34	150	T (0.78)	C (0.22)	0.64
*13738*	17	0.07	158	C (0.96)	T (0.04)	1.00
*18241*	17	0.02	159	G (0.99)	C (0.01)	1.00
*34117*	17	0.34	158	C (0.78)	T (0.22)	1.00
*4106*	18	0.02	159	C (0.99)	G (0.01)	1.00
*24550*	19	0.37	159	A (0.75)	G (0.25)	0.52
*25627*	19	0.49	159	G (0.55)	T (0.45)	0.52
*27998*	19	0.34	159	G (0.79)	A (0.21)	0.81
*13161*	20	0.48	159	G (0.59)	A (0.41)	0.62
*16861*	21	0.49	159	G (0.56)	A (0.44)	0.11

### Statistical analysis

The genetic data were used to assess admixture, population structure, and relatedness between individuals. Admixture was analyzed using STRUCTURE (Pritchard et al. 2000), with the following settings: For each individual, sampling habitat was taken as an informative prior for the final assignment of admixture proportion; each run consisted of 250,000 burn‐in cycles followed by 50,000 sampling cycles. Because the number of putative populations (K) was three, we tested for admixture between *K* = 2–4 populations. As a measure of differentiation, we used Weir and Cockerham's (1984) overall measure of *F*_st_ (rather than the pairwise Balding–Nichols *F* [Balding and Nichols 1995; ] or the individual loci's *F*_st_), computed on the full 32 loci dataset, using the R package hierfstat (Goudet 2005). In order to ascertain whether the sample size, both in terms of individuals and SNPs, was sufficient to yield a robust measure of *F*_st_, we simulated a population of 4500 individuals and 3150 SNPs. This population was divided into three subpopulations (1500 individuals for each subpopulation), which represented an island model with migration at equilibrium; changing the migration rate allowed us to change the resulting overall *F*_st_. From this population, we sampled 32 SNPs and 159 individuals to match our sample and calculated the overall *F*_st_. We repeated this sampling 10,000 times to generate a distribution of *F*_st_ values that was compared with the observed *F*_st_ in the empirical data. These simulations were conducted using simuPOP (Peng and Kimmel 2005) and hierfstat (Goudet 2005).

Population structure was analyzed following Astle and Balding (2009). Briefly, the data were transformed in a numeric matrix *M* where the genotype of each SNP was converted into a numerical variable (0/1/2) depending on the number of minor alleles present in each individual's genotype. This numeric variable was then transformed to a standard score. A kinship matrix *K* was calculated as *MM* ^*T*^ */2n*, where *2n* is twice the number of SNPs in use and *M* ^*T*^ is the transpose of *M*. The eigenvectors of the matrix *K* are the principal components describing population structure. In practice, the Astle and Balding (2009) approach calculates kinship as in the method of moments described by Ritland (1996), which has the advantage of providing an unbiased kinship estimate.

Pairwise relatedness was calculated using the R package *related* (Pew et al. 2015), which provides seven measures of coancestry, corresponding to twice the kinship coefficient calculated by the Astle and Balding (2009) method. The seven coancestry methods were those of Milligan (2003), Li et al. (1993), Lynch and Ritland (1999), Queller and Goodnight (1989), Ritland (1996), Wang (2002, 2007). This library also allows testing whether individuals within each putative population are more closely related than would be expected under the null hypothesis of all individuals belonging to one single population. This test was conducted by permuting (2500 times) the individuals (genotypes) across groups (keeping group size fixed), to obtain a “null” distribution of average pairwise coancestry values for each group. The actual average pairwise coancestry for each group can then be compared to this null distribution, and an empirical *P*‐value can be given to the hypothesis that the average pairwise coancestry within a group is not higher than the null generated by permutation. As we had three putative groups and seven different coancestry estimators, this resulted in 21 comparisons. Thus, we applied a Bonferroni correction to the resulting *P*‐values to account for multiple testing, with a new significance threshold of ~ 0.002.

### Data accessibility statement

The data underlying this publication have been deposited to Dryad: doi:10.5061/dryad.j5q12.

## Results

### Admixture

**Figure 1:**
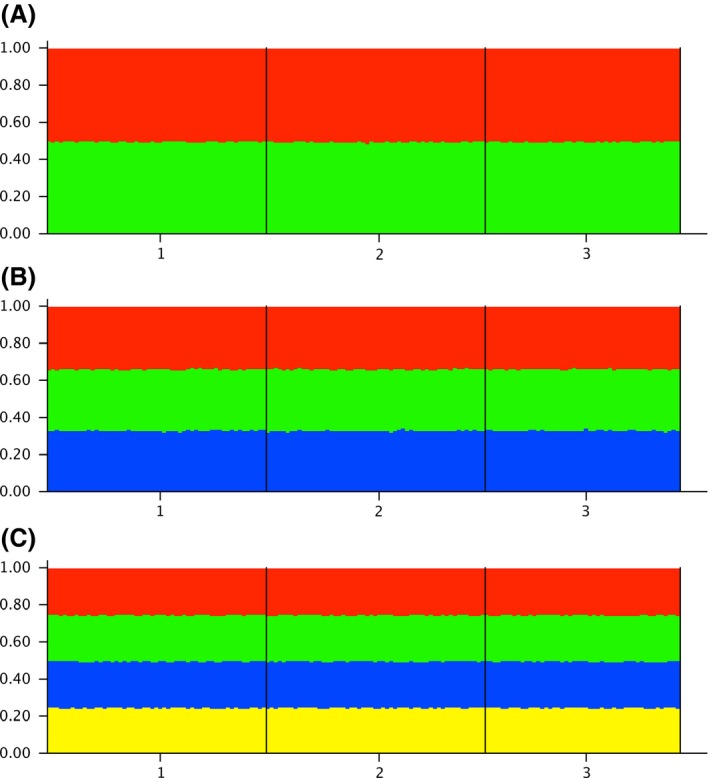
Results of admixture tests obtained with program STRUCTURE (Pritchard et al. 2000) assuming (A) two, (B) three, and (C) four putative clusters. All individuals in all three putative populations (1 = benthic, 2 = littoral, 3 = pelagic) are indicated to be equally admixed, and the admixture levels indicate an equal contribution of all three putative populations to each individual.

Our analysis revealed a state of complete panmixia, with each individual in the analysis showing to be a perfectly proportional mixture of all the possible putative populations, irrespective of how many populations (2, 3, or 4) the program STRUCTURE was trying to identify in the data (Fig. 1).

### Population structure

**Figure 2:**
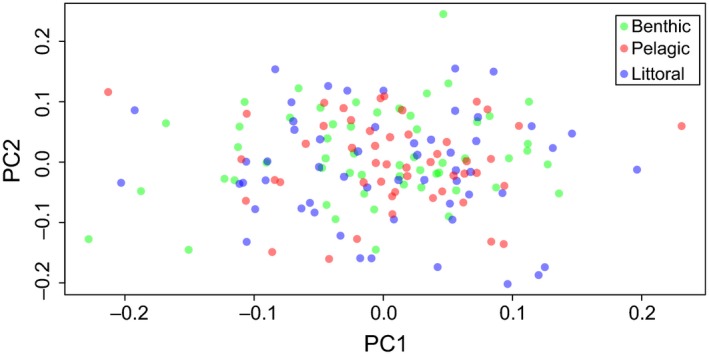
Analysis of population structure among three samples (littoral, benthic, and pelagic) of nine‐spined sticklebacks from a single lake based on principal component analysis of kinship matrix.

The degree of genetic differentiation among the three putative populations was low, with *F*_st _= 0.0002. Principal component analyses supported the lack of population structure in the data: The plot of the two first principal components of the kinship matrix did not reveal any clustering, suggesting that the sampled individuals came from the same population (Fig. 2). Comparison of the observed *F*_st_ in the data with the simulation results revealed that, for a population divided into three subpopulations with an *F*_st_ of ~ 0.1, our result was never part of the sampling distribution (Fig. S1A). This suggests that it is very unlikely that our data could have been sampled from a population with that level of *F*_st_ between subpopulations. On the other hand, when comparing the empirically derived *F*_st_ with a simulated population with three subpopulations having *F*_st_ ≈ 0.0025, the empirical *F*_st_ was not significantly different than what could be expected from a random sample from this population (empirical *P*‐value = 0.11; Fig. S1B). These results suggest that the lake population is genetically very weakly structured, most likely at a level no greater than *F*_st_ ≈ 0.0025.

### Pairwise relatedness

**Figure 3:**
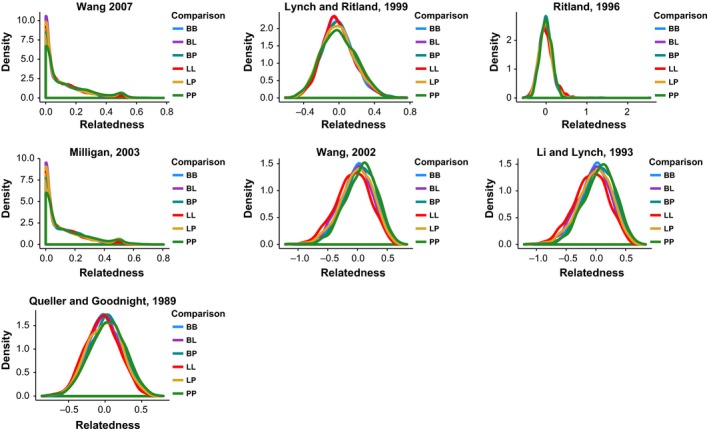
Comparisons of seven different pairwise coancestry coefficients within and between the three putative populations. The three populations are coded as B = benthic, L = littoral, and P = pelagic; thus, BB, LL, and PP indicate the distribution of the pairwise coancestry coefficient within putative populations, and BL, BP, and LP indicate between putative population comparisons.

The seven different methods of coancestry calculation showed a different spread of coancestry coefficients, depending on whether they were unbiased or constrained between zero and one (Fig. 3). However, irrespective of the estimate used, there was no pattern that distinguished the pairwise coancestries between pairs belonging to the same putative population and pairs belonging to different putative populations (Fig. 3). Testing whether the coancestry within each putative population was greater than expected by chance alone revealed that the actual within‐group relatedness was never significantly greater than what would be observed if the putative groups had been drawn at random from the whole population (in all tests, *P* ≥ 0.004, i.e., always greater than the Bonferroni‐corrected significance threshold).

## Discussion

Our analyses did not detect any form of population or kin structuring, suggesting that nine‐spined sticklebacks from the different habitats in the study lake were all members of a single panmictic population. While this may not be surprising given the small size and relatively young age of this postglacial lake, it is worth noting that sympatric speciation, or at least a strong within‐lake population structuring, is fairly common in fish (Kocher 2004; Stauffer and van Snick Gray 2004; Gante and Salzburger 2012; Ford et al. 2015; Seehausen 2015), even in postglacial habitats (Schluter 1996b; Hendry et al. 2013; McGee et al. 2013). This type of population structuring is typically associated with trophic niche differentiation between the divergent forms, for example, in the three‐spined stickleback (*Gasterosteus aculeatus*; Schluter 1993), a close relative of the nine‐spined stickleback. Three‐spined stickleback trophic morphs are known from several North American lakes (Lavin and McPhail 1986; Schluter 1993), but no such morphs have been described from the nine‐spined sticklebacks. This is in spite of the fact that the life‐history characteristics of this species potentially facilitate resource specialization and divergence (cf. Andersson et al. 2007). However, as genetic differentiation – and even speciation (Bickford et al. 2007) – is common even in the absence of any notable phenotypic differentiation (e.g., Conover and Schultz 1995), the early stages of habitat specialization and restricted gene flow are easily overlooked. In fact, this is what provided the impetus for this study.

In spite of the nine‐spined stickleback's circumpolar distribution – which testifies to the species' success in colonizing new areas – the nine‐spined stickleback is thought to be far less dispersive than the three‐spined stickleback (DeFaveri et al. 2011). This inference is based on the observation that the degree of genetic differentiation among nine‐spined stickleback populations exceeds that seen between three‐spined sticklebacks collected from the same localities (DeFaveri et al. 2011). Given this, as well as the evidence for habitat‐related genetic differentiation in other postglacially established fish populations (e.g., Schluter 1996b), it would not have been surprising to find at least weak genetic structuring among the fish collected from different habitats. However, all tests for such structuring failed to recover any indication of limited gene exchange between habitats. Hence, pending few potential caveats, the conclusion must be drawn that the fish in the study lake are one interbreeding population.

The lack of genetic differentiation among individuals from different habitats could also in theory be explained by a lack of statistical power to detect existing differentiation. However, we consider this possibility unlikely. Specifically, the sample sizes in terms of number of individuals within each habitat type were fairly large (≈ 50 individuals, 100 genes), and similar to what is generally used in population genetic investigations (e.g., Rieseberg et al. 2012). Admittedly, the number of SNP markers remaining for the final analyses was not exceedingly high. However, as they were truly independent (located across different chromosomes) and moderately polymorphic (average *H*_E _= 0.29), we were unlikely to be underpowered to detect low‐to‐moderate (*F*_ST_ ≈ 0.01) divergence. In fact, our simulations suggest that the statistical power to detect this level of differentiation was quite high.

Finally, the results of the kinship analyses are of note. The data limitations were clearly obvious in the fact that we did not obtain a consistent result from the different estimators. Unbiased estimators – such as Ritland's (1996) or Wang's (2002) – are more likely to provide unreliable results that are difficult to interpret, such as negative coancestry coefficients in the case of insufficient data. On the other hand, coefficients that are constrained between (0,1) are more likely to give upward‐biased estimates in case of poor data. Because we do not have a pedigree for the samples being investigated, we cannot estimate the performance of these estimators, although the most important factor in determining the reliability of these estimates seems to be the population relatedness composition, rather than the number of markers used (Csilléry et al. 2006). The fact that the distribution of coancestry coefficients was very similar between the three putative populations for all methods supports the conclusion that within‐lake genetic structure was absent or very weak.

In conclusion, the first genetic test of within‐lake differentiation in Fennoscandian sticklebacks provides no support for habitat‐related genetic divergence in the particular lake studied. This is in spite of the fact that the lake had distinct habitat and resource types. Sampling of replicate lakes, as well as in structurally and ecologically more heterogeneous lakes, would be needed to unravel whether the results apply more generally to freshwater populations of sticklebacks.

## Conflict of Interest

None declared.

## Supporting information


**Figure S1.** Results of simulations of population structure (*F*_ST_) assuming different levels divergence (A: *F*_ST_ = 0.1; B: *F*_ST_ = 0.0025) among three subpopulations as compared to the observed *F*_ST_ (vertical red line) in the empirical nine‐spined stickleback data.
