# Supplementary figures and images for: A test for within‐lake niche differentiation in the nine‐spined sticklebacks (Pungitius pungitius)
Source: Ecol Evol. 2016 Jun 14;6(14):4753–60. doi: 10.1002/ece3.2182 (PMC4979704; doi:10.1002/ece3.2182)

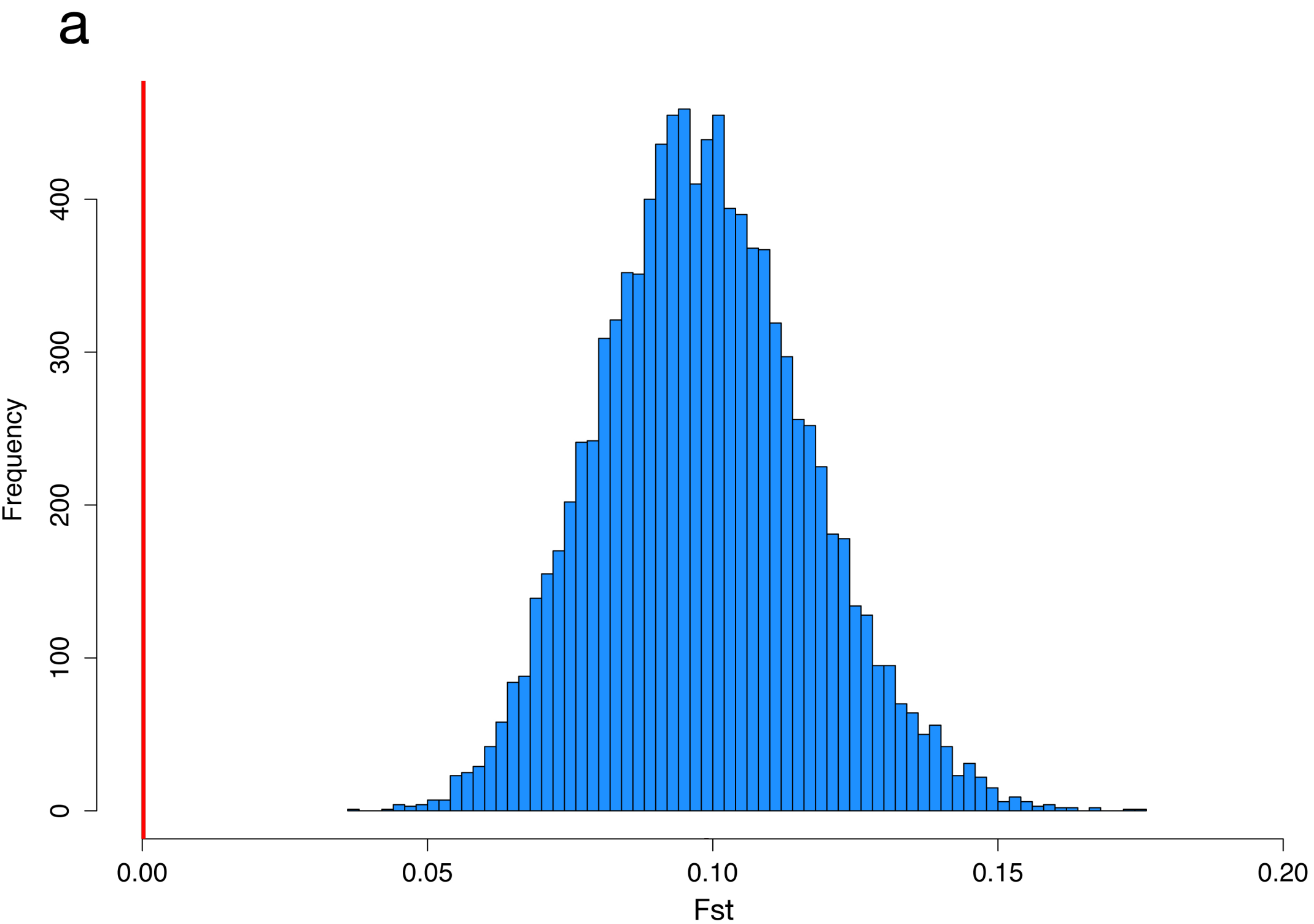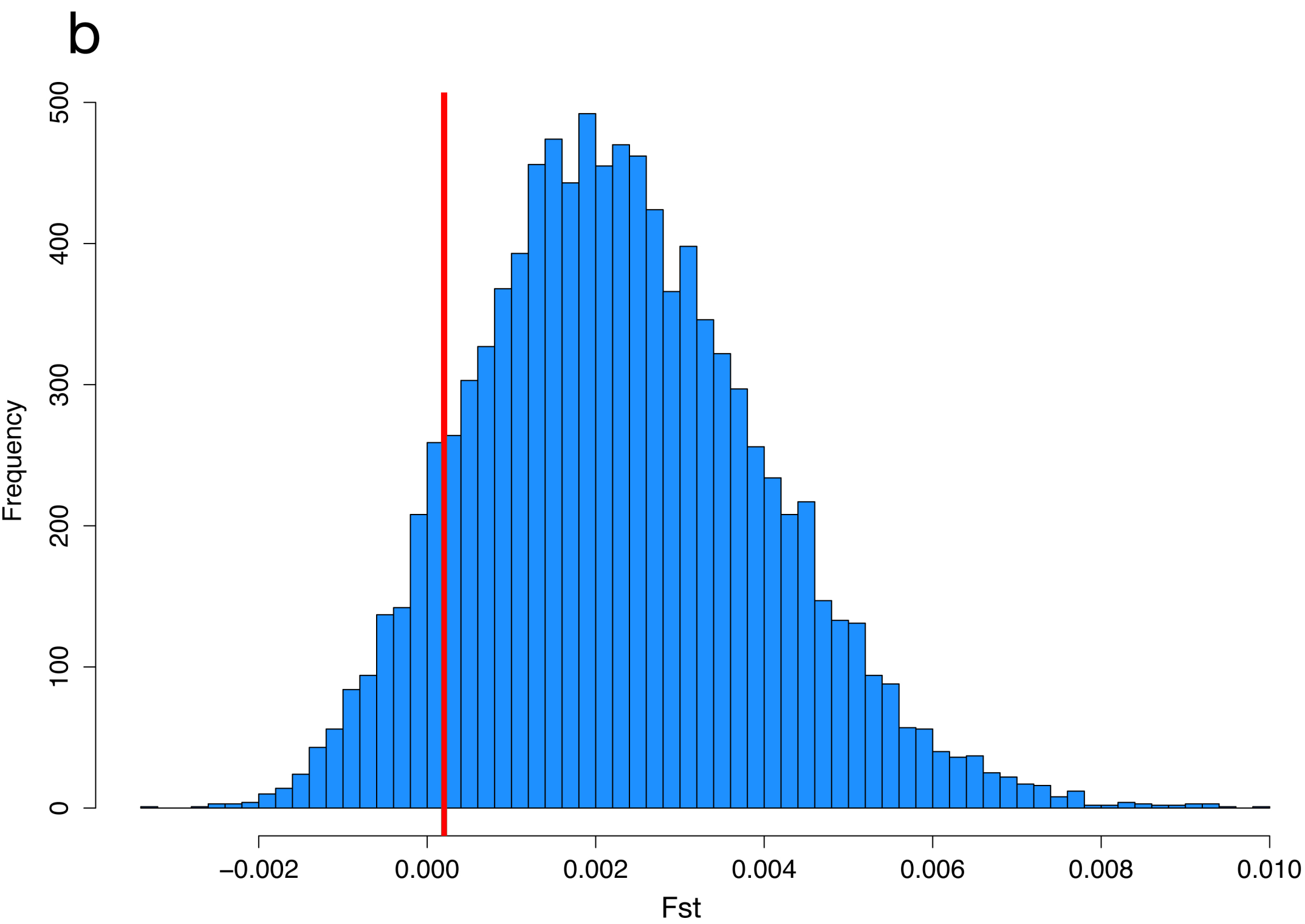

Supplement: Supplementary file 1 — Figure S1. Results of simulations of population structure (F ST) assuming different levels divergence (A: F ST = 0.1; B: F ST = 0.0025) among three subpopulations as compared to the observed F ST (vertical red line) in the empirical nine‐spined stickleback data. [file ECE3-6-4753-s001.pdf]
